# Development of animal model for Bisphosphonates-related osteonecrosis of the jaw (BRONJ)

**DOI:** 10.1186/s40902-015-0020-6

**Published:** 2015-07-25

**Authors:** Hyo-Won Jang, Jin-Woo Kim, In-Ho Cha

**Affiliations:** 1 Department of Oral and Maxillofacial Surgery, College of Dentistry, Yonsei University, Seoul, South Korea; 2 Department of Oral and Maxillofacial Surgery, Mok-dong Hospital, Ewha Womans University, Seoul, South Korea; 3 Department of Oral and Maxillofacial Surgery and Oral Cancer Research Institute, College of Dentistry, Yonsei University, 250 Seongsanno, Seodaemun-gu, Seoul 120-752 Republic of Korea

**Keywords:** Bisphosphonates, Osteonecrosis of the jaw, Steroid, Rat model

## Abstract

**Background:**

The aim of this study is to develop a rat model of bisphosphonates-related osteonecrosis of the jaw (BRONJ) that would be verified with clinical, radiological and histological examination, and to confirm the influence of concurrent bisphosphonates and steroids use upon the occurrence and aggravation of BRONJ.

**Methods:**

Twenty seven rats were divided into 3 groups; Saline group (I), Zoledronate group (II), Zoledronate and Dexamethasone group (III). Rats got weekly intraperitoneal injection for 4 times and extraction of left maxillary and mandibular 1st, 2nd molars were followed. Consecutive injections were performed, and blood sampling for measurements of C-terminal crosslinked telopeptide of type I collagen and tartrate-resistant acid phosphate 5b rats were performed at the time of 2, 4 and 8 weeks. And then, rats were sacrificed and evaluated clinically, radiologically and histologically.

**Results:**

12/18 (66.6 %) of experimental group were diagnosed as BRONJ. There was no significant difference in incidence between zoledronate alone group (ll) and concurrent use of zoledronate and dexamethasone group (lll).

**Conclusions:**

Concurrent use of bisphosphonates and steroids increase incidence of BRONJ compared to saline group (l). Zoledronate alone group (ll) and concurrent use of zoledronate and dexamethasone group (lll) shows same incidence of BRONJ. Based on this study, the rat treated with bisphosphonates and steroids can be considered a novel, reliable and reproducible model to understand pathology of BRONJ.

## Background

Bisphosphonates (BPs) are used to treat and prevent osteoporosis, bone metastasis of malignant disease, metabolic bone-disease(e.g. Paget’s disease, osteogenesis imperfecta, primary hyperparathyroidism) which causes secondary hypercalcemia, pathologic fracture [1–3].

BPs contain 2 phosphonates, same structure as pyrophosphate, and suppress the activity of osteoclasts, consequently decrease loss of bone volume [4, 5]. BPs are classified into 2 categories - Nitrogen containing BPs (pamidronate, alendronate, risedronate, zoledronate, etc.) and Non-nitrogen containing BP(clodronate, etidronate, etc.). Nitrogen containing BPs are hardly metabolized, and depress osteoclasts strongly, while Non-nitrogen containing BPs are metabolized quickly [6, 7].

In 2003, Marx first reported osteonecrosis of the jaw of patients, medicated by nitrogen containing BPs (pamidronate(Aredia®), zoledronate (Zometa®)) [8]. This report has triggered many discussions for pathogenesis, incidence, and treatment of BRONJ.

The American Society for Bone and Mineral Research(ASBMR) Task Force defines bisphosphonates-related osteonecrosis of jaw, as an area of exposed bone in the maxillofacial region that did not heal within 8 weeks in a patient who had been exposed to BPs, not to radiation therapy [9].

The incidence of BRONJ has not been defined, according to the research of ASBMR, BRONJ associated with oral administration of BPs for osteoporosis is estimated 1:100,000 to 1:10,000. However, it could be higher, depending on duration of therapy, and BRONJ associated with intravenous administration of high dose of BPs for cancer therapy is estimated 1:100 to 1:10 [9–12].

There are many risk factors for BRONJ – kind of BPs, administration route, patient age, pre-existing periodontal disease, invasive dental treatment, concurrent use of drugs [13, 14].

Kyrgidis reported that invasive dental treatment including tooth extraction is one of the significant factors for BRONJ [13]. Recently, ASBMR Task Force considers that systemic risk factors associated with cancer therapy, affect occurrence and aggravation of BRONJ, and concurrent use of chemo-regimen and steroids has synergic effects on BRONJ [14, 15].

Most of BRONJ patients were diagnosed with multiple myeloma, breast cancer, and prostate cancer. Furthermore, they were all injected with Nitrogen-containing BPs and steroids, such as dexamethasone intravenously. However, it has not been defined, interaction between systemic risk factors, concurrent medications, and occurrence and aggravation of BRONJ. Thus, the authors aimed to develop a rat model of BRONJ, and to confirm how concurrent use of BPs and steroids can affect occurrence and aggravation of BRONJ in this study.

## Methods

Twenty seven female Spraque-Dawley rats (250–300 g) were used and 54 sites (left maxilla and mandible). We followed the rules of Institutional Animal Care and Use Committee (IACUC), department of laboratory animal medicine, medical research center, Yonsei University, college of medicine.

**Fig. 1:**
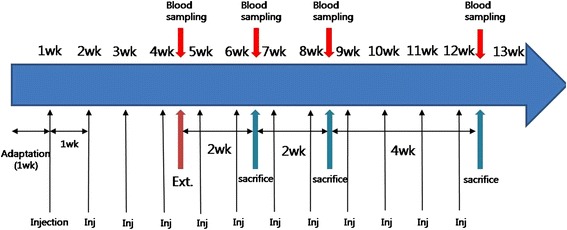
Time table of study is shown above. It takes 12 weeks, and 2,4,8 weeks after extraction, we sacrifice every 3 rats of control group, 2,2,5 rats of experimental group. We take blood samples at the same time

All rats(*n* = 27) are divided into 3 groups (Table 1). Saline group (l) was injected with normal saline intra-peritoneally. Zoledronate group (II) was injected with zoledronic acid 0.2 mg/kg intra-peritoneally. Zoledronate and dexamethasone group (lll) was injected with zoledronic acid 0.2 mg/kg, and dexamethasone 5 mg/kg intra-peritoneally, at the same time. Total study period takes 12 weeks (Fig. 1.).

**Table 1:** We used 27 rats, divided into 3 groups

Group number	Group information	Medication
1 (*n* = 9)	Control	Normal saline
2 (*n* = 9)	Zoledronic acid injection	Zoledronic acid 0.2 mg/kg
3 (*n* = 9)	Zoledronic acid + Dexamet hasone injection	Zoledronic acid 0.2 mg/kg + Dexamethasone 5 mg/kg

All rats were adapted for 1 week, according to the rules of Institutional Animal Care and Use Committee (IACUC). From the first day of study, for 4 weeks, medications are injected intravenously every 1 week by same experimentor. 3 days after 4th injection, left 1st, 2nd molars of maxilla and mandible were extracted. All extraction were done aseptically, under general anesthesia (Tiletamine/zolazepam®-milan, Italy, xylazine-Rompun® Bayer AG, Leverkusen, Germany). We used dental explorer, in procedure of peritomy, subluxation, luxation, extraction, and curettage, sequentially. After extraction, we injected meloxicam(Metacam®, Boehringer-Ingelheim, Germany), 10 mg/kg intra-peritoneally to control post-operative pain, according to IACUC.

2, 4, 8 weeks after extraction, every 3 rats of saline group (l) and 2,2,5 rats of each zoledronate group (ll) and zoledronate and dexamethasone group (lll) were sacrificed in CO_2_ asphyxiation chamber, taking blood samples infraorbitally at the same time. Blood samples are used on ELISA kit, to analyze CTX, TRACP 5b level. All the specimens were evaluated by clinical, radiological, histological examination.

### Clinical examination

All the specimens were reviewed to confirm incomplete oral mucosal coverage on necrotic alveolar bone and sequestra formation, then photographed, and preserved with 10 % buffered-formalin immediately.

### Radiological examination

All the specimens were radiographed by Plain X-ray with standard dental radiographic film.

(Voltage(kV) = 60, Current(mA) = 70, Exposure(s) = 0.08)

### Histological examination

All the specimens were decalcified with 10 % ethylenediaminetetraacetic acid (EDTA) for 1 week, and embedded to paraffin. After embedding to paraffin, maxilla and mandible were resected 4 μm thickness, anterio-posteriorly.

We used Hematoxylin & Eosin staining to see inflammatory cells, necrotic alveolar bone, and empty lacunae of osteoclasts. Also, we confirmed BRONJ with histologic features, calculating incidence of ONJ in experimental group.

### Laboratory & statistical examination

1 ~ 1.5 cc whole blood was taken from infra-orbitally, right before extraction of teeth and sacrifice, 2, 4, 8 weeks after extraction. Whole blood was centrifuged and applied to ELISA kit to evaluate CTX(C-terminal crosslinked telopeptide of type I collagen), TRACP 5b(Serum tartrate-resistant acid phosphate) level. CTX, Non-helical C-terminal of Type I bone tropocollagen, is a biomarker that evaluate the degree of type I collagen resorption and activity of osteoclasts [16]. We used CTX to evaluate the relation between osteonecrosis and laboratory finding. Serum tartrate-resistant acid phosphate 5b (TRACP 5b) is a novel marker for monitoring anti-resorptive therapy in patients using BPs [17, 18]. Herein, the authors used TRACP 5b as well as CTX, as the evidence for occurrence of BRONJ.

#### Bivariate analysis (Independant *t*-test)

We did independant *t*-test (Mann–Whitney test), because of small number of animal groups to find TRACP 5b can distinguish between normal and BRONJ group with statistical significance.

#### Linear mixed test

Linear mixed test shows group & time effects for TRACP 5b regarding BRONJ risk prediction.

## Results

### Clinical examination

In experimental group(group 2, 3), 12/18 rats(66.6 %) showed osteonecrosis of jaw.

**Fig. 2:**
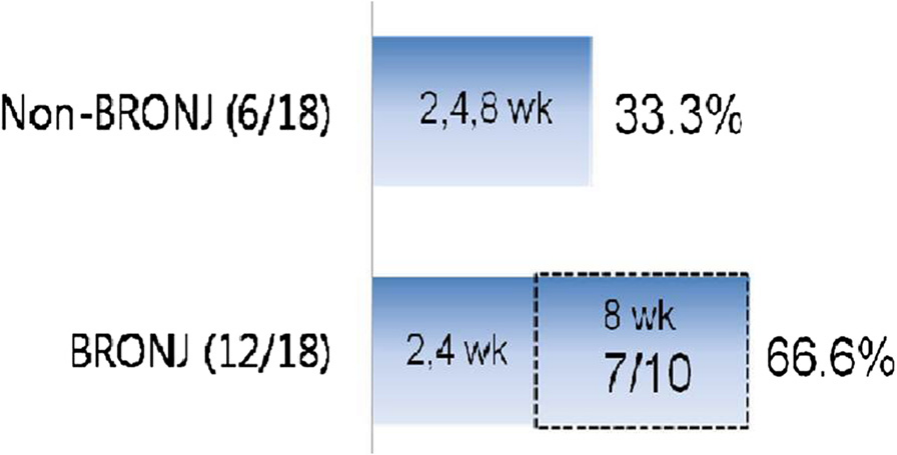
Incidence of ONJ lesion(%), experimental group

Seven of 10 rats, that were sacrificed 8 weeks after extraction, showed osteonecrosis of jaw, relatively high incidence of BRONJ(70 %). It explains that injection period can be a risk factor for BRONJ (Fig. 2).

**Fig. 3:**
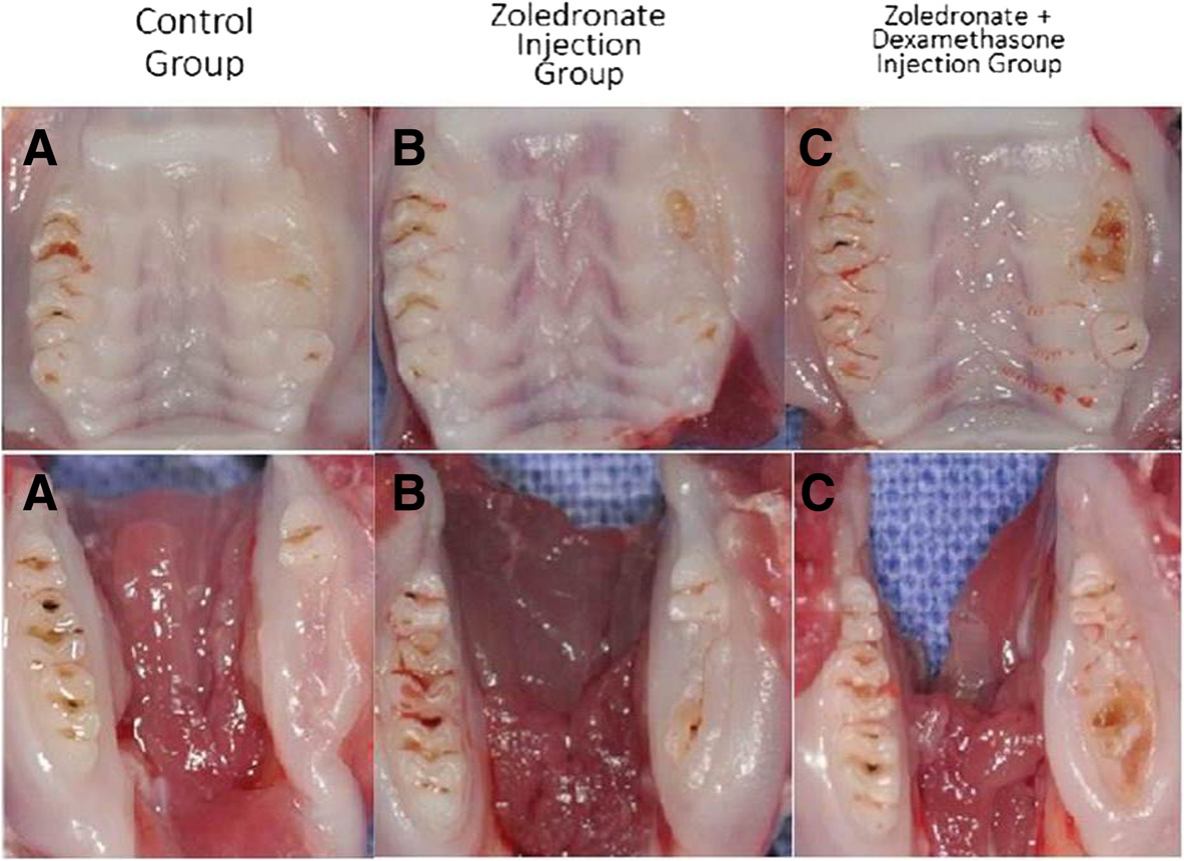
Representative photographs of gross clinical appearance of maxillary & mandibular ridge in rats(All sacrificed 8 weeks after extraction). **a** Control group shows complete healing of oral mucosa covering extraction site. **b** Z group shows incomplete healing of oral mucosa, showing exposed, necrotic, alveolar bone. **c** Z+D group shows incomplete healing of oral mucosa, showing definitely wide, exposed necrotic alveolar bone

We compared clinical appearances of maxillary & mandibular ridge in rats. Control group shows complete healing of oral mucosa (Fig. 3a). Zoledronate injection group(Z group) and Zoledronate & Dexamethasone injection group(Z+D group) show incomplete healing of oral mucosa, similar incidence but, different in size of exposed, necrotic alveolar bone (Fig. 3b and c).

### Radiological examination

**Fig. 4:**
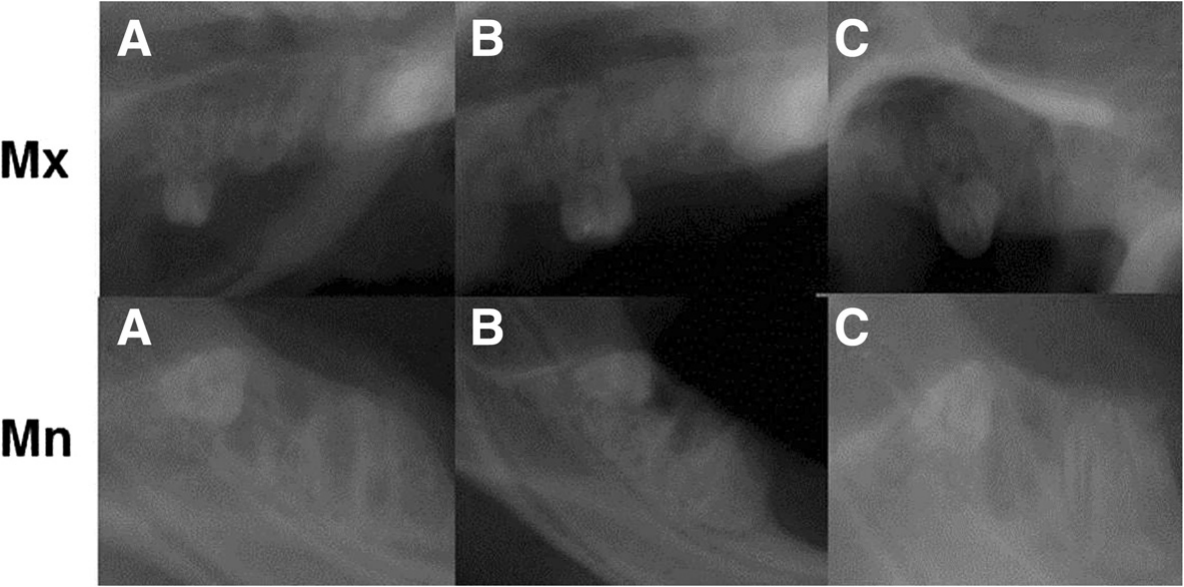
Representative radiograph of extraction sites on maxillary & mandibular ridge in rats(All sacrificed 8 weeks after extraction). **a** Linear opaque radio-density around extraction socket (control group). **b** Mottled trabecular pattern around extraction socket (Z group). **c** More definite, wider mottled trabecular pattern around extraction socket (Z+D group)

Control group shows complete healing of jaw bone - linear opaque radio-density around extraction socket (Fig. 4a). Experimental group shows incomplete healing of jaw bone - mottled trabecular pattern around extraction socket, more definite in Z+D group (Fig. 4b and c).

### Histological examination

**Fig. 5:**
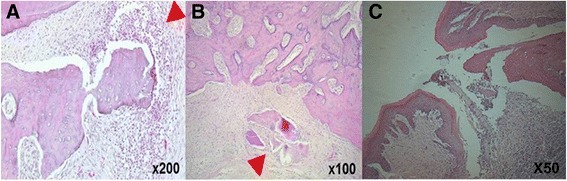
Representative histologic features of extraction sites(All sacrificed 8 weeks after extraction). **a** Inflammatory cells(neutrophils) surround necrotic alveolar bone with empty lacunae (Z group, Mn, ×200, sacrificed 8 weeks after extraction). **b** Sequestra formation apart from alveolar bone with empty lacunae (Z group, Mn, ×100, sacrificed 8 weeks after extraction). **c** Ulcerative overlying mucosa invaginates into extraction socket, and necrotic bone with empty lacunae on the surface of exposed alveolar bone (Z+D group, Mx, ×50, sacrificed 8 weeks after extraction)

All specimens in control group show complete healing of extraction sockets. Experimental group shows incomplete healing of extraction site. There were inflammatory cells surrounding necrotic alveolar bone with empty lacunae (Fig. 5a), and sequestra apart from alveolar bone (Fig. 5b). Ulcerative overlying mucosa invaginates into extraction socket (Fig. 5c).

### Laboratory & statistical examination

**Fig. 6:**
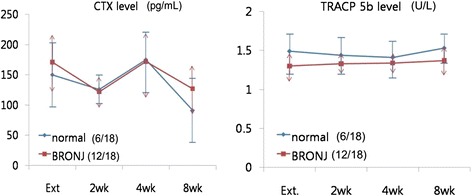
CTX shows no tendancy neither between normal and BRONJ group, nor within each group. TRACP 5b consistently shows lower value of BRONJ group than normal healing group, although time and TRACP 5b level are not inversely proportional

Here is the laboratory result (Fig. 6). TRACP 5b shows more reliable value to indicate BRONJ than CTX level - TRACP 5b consistently shows lower value of BRONJ group than normal healing group.

### Bivariate analysis (Independant *t*-test)

We did independant *t*-test (Mann–Whitney test) because of small number of animal groups.

In this test, TRACP 5b can distinguish normal and BRONJ group with statistical significance(*P* value <0.05) (Table 2). TRACP 5b has lower diurnal variability than CTX, although time and TRACP 5b level are not inversely proportional.

**Table 2:** TRACP 5b can distinguish between normal and BRONJ group with statistical significance

CTX	Non-BRONJ (6/18)	BRONJ (12/18)	*P* value
	Mean(pg/mL)	Mean(pg/mL)	
Baseline	150.63 ± 50.69	171.36 ± 45.05	0.23
2 weeks	126.24 ± 21.97	122.79 ± 24.58	0.87
4 weeks	175.13 ± 48.28	172.71 ± 40.78	0.90
8 weeks	91.69 ± 53.30	127.53 ± 38.00	0.12
TRACP 5b	Non-BRONJ (6/18)	BRONJ (12/18)	*P* value
	Mean(U/L)	Mean(U/L)	
Baseline	1.49 ± 0.19	1.30 ± 0.07	**0.00**
2 weeks	1.44 ± 0.18	1.33 ± 0.05	**0.01**
4 weeks	1.42 ± 0.17	1.34 ± 0.04	**0.07**
8 weeks	1.53 ± 0.16	1.37 ± 0.12	**0.03**

(*P* value < 0.05)

### Linear mixed test

Linear mixed test shows group & time effects for TRACP 5b regarding BRONJ risk prediction. Here is the equation considering ‘Effect factor’ (e^SE^) (Table 3).

**Table 3:** Linear mixed test equation and effect factor

	Estimated measures (ES)	95 % CI	*P* value
BRONJ	0.18 ± 0.05	0.08 ± 0.28	**0.001**
Time	0.02 ± 0.01	0.003 ± 0.05	0.023
BRONJ × Time	−0.02 ± 0.02	−0.05 ± 0.01	0.229

Effect factor = e^SE^

***y*** **= X** ***β*** **+** ***Z*** **u +** ***ε***

y: TRACP 5b measures

*β* : Fixed effects

*u*: Random effects

*ε*: Random error

It concludes that

1. TRACP 5b has e0.18 effect to distinguish BRONJ & Non-BRONJ group, with statistical significance. (*P* value = 0.001)
2. TRACP 5b has e0.02 effect to predict incidence of BRONJ with time change, without statistical significance. (*P* value = 0.023)
3. It is possible to use TRACP 5b to diagnose BRONJ to a certain degree.

## Discussion

There are many studies to investigate interaction between bisphosphonates and other drugs – steroids [15, 19], vitamin D [20], on incidence of BRONJ.

The concurrent use of bisphosphonates and steroids has definite clinical relevance with BRONJ, as many BRONJ patients being treated for multiple myeloma, breast cancer receive both drugs [13]. The purpose of this study focused on development of rat model of BRONJ to confirm how concurrent use of BPs and steroids can affect occurrence and aggravation of BRONJ.

We used equivalent dose of zoledronate and dexamethasone. For example, multiple myeloma patients take zoledronic acid intravenously, 1.6 × 10 ^−4^ g/kg/week. We injected 2.0 × 10 ^−4^ g/kg/week intraperitoneally, assuming that effective dose of zoledronic acid, injected peritoneally can be lower than that of intravenous injection. It resulted in increased concentration and frequency of bisphosphonates injection, more than usual clinical use for cancer patients. As a result, we have 66.6 % of BRONJ rats, much higher than human incidence of BRONJ, 1–10 %.

In control group, we observed that new bone formation starts 2 days after extraction, new bone fills extraction socket completely 2 weeks after extraction, as previous studies report [21, 22].

In our animal model, it appeared that bisphosphonates alone and concurrent use of bisphosphonates and steroids has similar incidence of BRONJ in clinical, radiological, histological examination.

It can be explained in two ways.

- First, root remnants may affect healing mechanism of BRONJ. We used dental explorer extracting molar teeth, in process of subluxation, luxation, extraction, curettage. Complete extraction and curettage makes bigger bone defect than incomplete extraction. Rat molar has more divergent roots than those of human, resulting in more chances to leave root remnants. Badros reported that patients with pamidronate and dentoalveolar surgery together have 7 times higher incidence of BRONJ than those who have pamidronate alone [23]. That explains bigger bone defect increases incidence of BRONJ.
- Second, BRONJ patients are usually older and take many kinds of drugs than rats. Mean life of Spraque-Dawley rat is 24 months, 96 weeks. We used 8 week-rats, it takes 12 weeks to finish this study. So they are 20 weeks-old and still adolescent. Zoledronate alone or concurrent use of zoledronate and steroid may make no difference to immune system of adolescent rats.

Steroids may reduce post-operative inflammation to help healing, but in our study, cumulative doses of steroids can modify the bony and mucosal reponse to delay healing, causing avascular necrosis [24], increasing BRONJ risk [19, 25, 26], as previous studies report.

There are many biomarkers to diagnose BRONJ – CTX [16], NTX level, TRACP 5b [17], RANKL/OPG ratio, etc. [27].

We chose CTX and TRACP 5b to confirm the diagnosis of BRONJ. TRACP 5b has two isotypes- Type 5a & Type 5b. Most of TRACP 5b are type 5b, they exist in osteoclasts. So we can analyze activity and numbers of osteoclasts, analyzing TRACP 5b quantitatively. Besides, TRACP 5b is not affected by liver and kidney function, it has little diurnal variability than CTX.

There were many reports using bone turn-over markers including TRACP 5b [28], but not in field of BRONJ, that represents much to this study.

In this model, TRACP 5b shows more reliable value to indicate BRONJ than CTX level - TRACP 5b consistently shows lower value of BRONJ group than normal healing group, although time and TRACP 5b level are not inversely proportional. There are many bone turn-over markers including TRACP 5b, but clinical use is not established yet. There should be more research for clinical use of TRACP 5b, predicting possibility of BRONJ incidence, staging and managing of BRONJ patients.

## Conclusion

We established successful animal model for BRONJ, and obtained following results.

1. Concurrent use of bisphosphonates and steroids increase incidence of BRONJ.
2. It has little chance of spontaneous BRONJ, resulting from concurrent use of bisphosphonates and steroids only, surgical stimulus, such as extraction plays important role as trigger factor, increasing incidence of BRONJ.
3. Based on this study, the rat treated with bisphosphonates and steroids can be considered a novel, reliable and reproducible model to understand better pathology of BRONJ.
4. TRACP 5b can be a reliable biomarker to confirm BRONJ.
